# Motor Nerve Transfers in Complete and Incomplete Brachial Plexus Injuries: A State-of-the-Art Review

**DOI:** 10.3390/neurolint18060103

**Published:** 2026-05-25

**Authors:** Leonardo Bradaschia, Christian Heinen

**Affiliations:** 1 Department of Neuroscience “Rita Levi Montalcini”, University of Turin, 10126 Turin, Italy; 2 Neurosurgery Unit, “Città della Salute e della Scienza” University Hospital, University of Turin, 10126 Turin, Italy; 3 Peripheral Nerve Unit Nord, Christliches Krankenhaus Quakenbrück GmbH, 49610 Quakenbrück, Germany; c.heinen@ckq-gmbh.de

**Keywords:** traumatic injuries, nerve graft, neurotization, coaptation, nerve transfer

## Abstract

Brachial plexus injuries are challenging conditions. Over the past decades, nerve transfer surgery has progressively evolved from proximal nerve reconstruction toward selective distal neurotization strategies, considerably expanding the possibilities for functional restoration. As the number of described donor–recipient combinations has increased, the literature has become increasingly fragmented, often focusing on isolated techniques or specific functional targets. The aim of the present study was to provide a comprehensive state-of-the-art overview of currently available motor nerve transfer strategies for upper-limb reinnervation in BPI. A literature review was conducted according to PRISMA guidelines using PubMed/MEDLINE, Embase, Cochrane Library, Scopus, and Web of Science databases. Studies concerning motor nerve transfers for upper-limb reconstruction were systematically reviewed and categorized according to recipient nerve and functional target, including shoulder function, scapular stabilization, elbow flexion and extension, wrist and finger extension, wrist and finger flexion, intrinsic hand function, and extraplexal donor nerve reconstruction. A total of 250 studies met the inclusion criteria. Both intraplexal and extraplexal donor strategies were identified for most reconstructive targets. Intraplexal distal nerve transfers currently represent the preferred approach whenever feasible because of shorter reinnervation distances and more predictable outcomes. Extraplexal donors, including the spinal accessory, intercostal, contralateral C7, and phrenic nerves, remain essential in complete BPIs and root avulsion injuries. Despite substantial advances, restoration of intrinsic hand function and reliable distal reinnervation remain major reconstructive challenges. Motor nerve transfers represent an increasingly versatile and function-oriented reconstructive strategy that should be tailored to the individual injury pattern, available donor nerves, and functional priorities.

## 1. Introduction

Brachial plexus injuries (BPIs) have a profound impact on both patients and the healthcare system, often resulting in physical impairments, poor functional outcomes, and high levels of disability [1]. These injuries are particularly common following motor vehicle accidents. Although the exact annual incidence of BPI is difficult to determine, the increasing number of survivors of high-energy trauma has led to a corresponding rise in cases [2].

Since the beginning of the twentieth century, advances in the understanding of nerve regeneration and surgical techniques have led to the development and refinement of nerve transfers. A nerve transfer involves coapting a healthy, expendable donor nerve (mostly defined portions, rarely the whole nerve)—sacrificing parts of the muscle it originally innervates—to a denervated recipient nerve, thereby restoring function to the target muscle.

The first reported nerve transfers were attempted in 1903 by Harris and Low, who coapted the distal stump of a ruptured C5 root to the healthy C6 root [3] and by Balance in the same year, who performed a spinal accessory nerve (SAN)–to–facial nerve transfer [4]. However, it was Tuttle in 1913 who first applied this technique to the treatment of BPI, specifically for a ruptured upper trunk [5]. Over the years, multiple attempts have been made to identify the ideal donor nerve for the reconstruction of each injured nerve and its associated function, leading to an increasing number of nerves being utilized and to modifications of previously established approaches.

According to the type of nerve involved, nerve transfers can be classified as motor or sensory. Motor transfers aim to restore movement, whereas sensory transfers aim to recover at least protective sensation, thereby preventing skin ulcerations and contributing to the control of neuropathic pain [6].

Coaptation can be performed using different techniques. End-to-end anastomosis seems to be the most successful method for motor restoration. End-to-side anastomosis involves coapting the proximal end of an injured nerve to the side of a healthy nerve after creating an epineurial, and preferably perineurial, window, allowing lateral sprouting of sensory axons [7]. However, motor axons require an injury (crush or axotomy) to sprout into an end-to-side–coapted damaged nerve [8] making this technique largely ineffective for motor reinnervation. Reverse end-to-side, or “supercharge,” end-to-side anastomosis consists of transecting a healthy nerve and coapting it to the side of a damaged nerve, thereby preventing muscle atrophy by “babysitting” the target muscles until regeneration from the proximally injured recipient nerve occurs [9].

Although a recent book has comprehensively addressed the topic of nerve transfers [10], a single article providing a complete state-of-the-art overview of all currently available motor nerve transfer strategies is still lacking. Furthermore, such monographic works do not fully incorporate emerging evidence from cadaveric studies or preliminary clinical experiences reported as isolated case reports, which may nonetheless offer valuable insights into the ongoing evolution of surgical techniques.

The aim of the present study is to summarize the current state of the art of motor nerve transfer techniques in the surgical field sorted systematically by the respective movement to achieve.

## 2. Materials and Methods

To assess all potential strategies involving motor nerve transfers for the management of BPIs, a literature review was conducted in accordance with the Preferred Reporting Items for Systematic Reviews and Meta-Analyses (PRISMA) guidelines. Institutional ethics approval was not required.

### 2.1. Search Criteria

A comprehensive literature search was performed in February 2026 to identify all full-text English-language studies concerning motor nerve transfers for upper-limb reconstruction. The following databases were searched: PubMed/MEDLINE, Embase, Cochrane Library, Scopus, and Web of Science.

Search terms included code words or their combination with associated Boolean operators including: “Brachial Plexus injury” OR “Brachial Plexus avulsion” AND “Nerve transfer” OR “Axillary nerve” OR “Musculocutaneous nerve” OR “Radial nerve” OR “Spinal accessory nerve” OR “Suprascapular nerve” OR “Long thoracic nerve” OR “Median nerve” OR “Ulnar nerve” OR “Phrenic nerve”. In addition to the automated search, a manual review of the references of all retrieved articles was performed to identify any additional relevant studies.

The aim of the search was to identify all studies concerning motor nerve transfers for upper-limb reinnervation and to classify the different transfers according to the functional target (e.g., shoulder abduction, elbow flexion, finger extension).

### 2.2. Selection Criteria

The inclusion criteria were as follows:

- Articles published in English;
- Case reports, case series, randomized controlled trials, cadaveric feasibility studies, and observational cohort studies;
- Studies specifically addressing the management of BPIs through nerve transfer procedures and reporting at least one motor nerve transfer, either applied clinically or investigated in cadaveric studies.

The exclusion criteria were as follows:

- Studies concerning sensory nerve transfers, lower-limb nerve transfers, or tendon transfer;
- Studies addressing direct nerve repair or nerve grafting of the affected nerve;

### 2.3. Outcomes

After removal of 148 duplicate records, 383 potentially relevant articles were identified. Screening based on title and abstract led to the exclusion of 47 articles because the full text was unavailable or the manuscript was not written in English. An additional 86 articles were excluded because they addressed unrelated topics. Ultimately, 250 studies met the inclusion criteria.

The included studies were categorized into seven main groups according to their primary functional target:

- Shoulder external rotation and abduction
- Winging scapula
- Elbow, wrist and finger extension
- Elbow flexion
- Hand pronation, wrist and fingers flexion
- Intrinsic hand musculature
- Extraplexal nerves

The “shoulder external rotation and abduction” group included nerve transfers targeting both the suprascapular and axillary nerves, whereas the “extraplexal donor nerves” group included studies involving the phrenic and SAN as donor nerves. For all nerve targets except those included in the latter group, both intraplexal and extraplexal donor nerves were evaluated.

Some studies reported multiple nerve transfer procedures and were therefore included in more than one category within this review.

Specifically, the studies were distributed as follows:

- 37 for the first group (18 involving the suprascapular nerve and 19 involving the axillary nerve)
- 9 for the second group
- 67 for the third group
- 81 for the fourth group
- 38 for the fifth group
- 7 for the sixth group
- 11 for the seventh group (9 involving the phrenic nerve and 2 involving the spinal accessory nerve)

**Figure 1:**
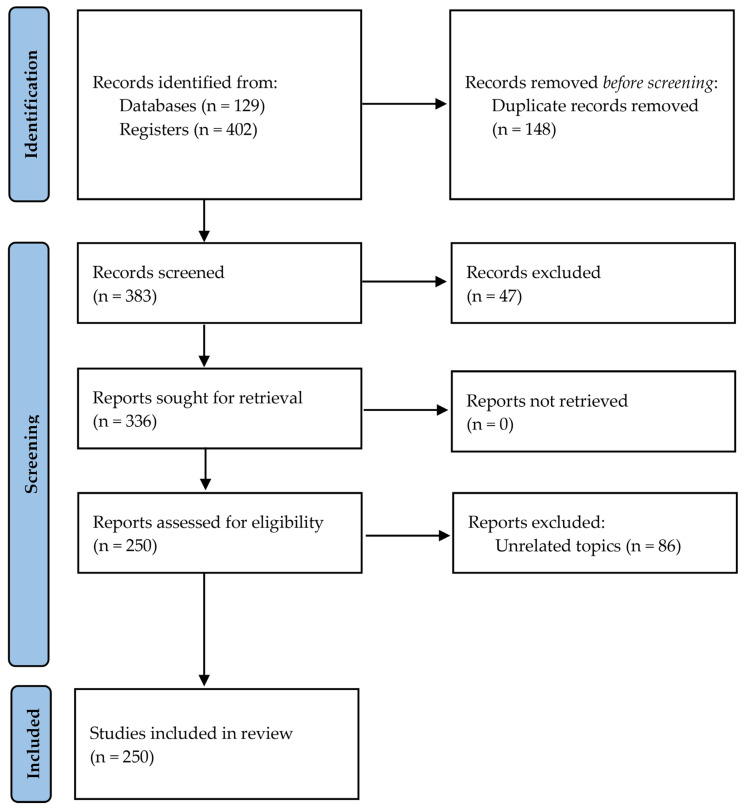
PRISMA diagram showing the article selection in the literature review.

A PRISMA diagram is provided in Figure 1, and the list of included articles is summarized in Table 10.

## 3. Shoulder External Rotation and Abduction

### 3.1. Transfer to the Suprascapular Nerve

The suprascapular nerve (SSN) innervates the supraspinatus and infraspinatus muscles, contributing to shoulder abduction and external rotation, respectively [11]. This nerve is commonly affected in injuries involving the C5 and C6 nerve roots or the upper trunk of the brachial plexus. In the absence of surgical intervention, patients may develop shoulder subluxation or dislocation, internal rotation contractures, and glenohumeral dysplasia.

#### 3.1.1. Intraplexal Nerve Transfer

In 2015, Goubier and Teboul first proposed the transfer of the dorsal scapular nerve (DSN) to the suprascapular nerve (SSN), using the terminal branch of the DSN to the rhomboid muscles, in a cadaveric study [12]. The procedure was successfully performed clinically the following year [13]. In 2020, the same group reported their long-term results in eight patients, demonstrating the effectiveness of the procedure while also highlighting its limitations compared with SAN transfer. Based on these findings, the authors recommended reserving this technique for patients without a functional SAN available for transfer or when the SSN is not accessible in the cervical region [14]. Similar results were reported by Augustine et al. in 2017 in a patient with Erb’s palsy [15]. The rationale underlying this nerve transfer lies in the fact that the SSN is commonly involved in upper brachial plexus injuries, whereas the DSN, although classically described as originating from the C5 root, frequently arises from C4 or occasionally C3 [16], thereby explaining its preservation in most complete BPIs.

Another option for reconstruction of the SSN in C5–C6 BPI with an intact C7 root was described by Cambon-Binder et al. in 2022. In this technique, a fascicle of the pectoralis major muscle arising from the C7 root is harvested as the donor nerve [17], thereby preserving the SAN and, consequently, the trapezius muscle for potential future tendon transfer if needed. Although the reported outcomes are favourable compared with other extraplexal nerve transfers [18], his approach is less commonly used than alternative nerve transfer techniques.

In 2010, David et al. proposed, in an anatomical study, the feasibility of using the pectoral nerves to reinnervate both the SSN and the axillary nerve [19]. They concluded that the middle and inferior branches of the pectoral nerves could represent suitable donor nerves for the axillary nerve and a potential source of reinnervation for the SSN; however, to date, no clinical case reports have been published.

#### 3.1.2. Extraplexal Nerve Transfer

In the early 1980s, several authors described the possibility of harvesting the SAN as an extraplexal donor for nerve transfer [20,21,22,23] in cases of total BPI or brachial plexus avulsion (BPA). Initially employed for multiple targets, the SAN has more recently been used primarily for reconstruction of the SSN. This preference is due to the close proximity of the donor and recipient nerves, which allows for a direct end-to-end neurorrhaphy, thereby avoiding the need for nerve grafting [24,25]. In addition, shoulder elevation (i.e., shrugging) through activation of the trapezius muscle is a natural synergistic movement when attempting to raise the arm, facilitating rapid and intuitive re-education of the nerve transfer.

Harvesting of the SAN may be performed through either an anterior [26] or posterior approach [27,28]. The anterior approach is more commonly associated with exploration of the injured brachial plexus and is therefore particularly useful in cases of total BPI or BPA. In such cases, a classical transverse approach may be used, as well as the extended approach described by Bertelli and Ghizoni [26]. However, when the SSN is retracted and exposure of the suprascapular notch is required, the more extensive approach described by Bertelli and Ghizoni [29] becomes necessary. In extreme cases, the contralateral SAN has been used, as reported by Zermeño-Rivera and Gutiérrez-Amavizca; however, outcomes were poor, likely due to the excessive length of the nerve graft required (a 50-cm cutaneous antebrachial graft) [30].

In 2009, Durand et al. proposed the first intercostal nerve (ICN) as a donor for the SSN instead of the SAN, demonstrating its feasibility in a case report [31]. The main advantage of this technique is that the first ICN is a purely motor nerve, and its axonal count is highest in the paravertebral region, where it is harvested, thereby allowing tension-free coaptation.

Finally, Gu et al. popularized the use of the phrenic nerve (PH) as a donor nerve [32] and several authors have reported the usefulness of PH transfer to the SSN [33,34,35], considering the PH superior to the SAN because it avoids denervation of part of the trapezius muscle [35]. On the other hand, the SAN is a purely motor nerve, and its harvesting does not compromise respiratory function. All data are summarized in Table 1.

### 3.2. Transfer to the Axillary Nerve

The axillary nerve (AN) is one of the most commonly injured nerves around the shoulder, typically as a result of iatrogenic injury, shoulder dislocation, or direct trauma. It innervates both the deltoid (anterior branch) and teres minor (posterior branch) muscles. The deltoid contributes to arm abduction, lateral rotation, and partially to extension, while the teres minor stabilizes the glenohumeral joint and helps maintain the humeral head within the shoulder capsule [36]. Finally, the AN provides cutaneous innervation to the inferior lateral deltoid region, commonly referred to as the “regimental badge” area [37].

Owing to its unique anatomy, multiple surgical approaches are available to access different portions of the AN, facilitating the use of a variety of donor nerves for transfer when direct reconstruction is not feasible.

#### 3.2.1. Intraplexal Nerve Transfer

In 2003, Witoonchart et al. demonstrated in a cadaveric study the feasibility of transferring a branch of the radial nerve to the long head of the triceps muscle to the anterior division of the AN for deltoid reinnervation [38]. Later that same year, this concept evolved into the technique now commonly referred to as the “Somsak procedure” [39]. The main difference between this technique and that described by Bertelli in 2004 lies in the surgical approach: the Somsak procedure is performed through a posterior approach, whereas Bertelli described an anterior approach [40]. Both techniques have subsequently been modified, with branches to the medial head of the triceps and to the anconaeus muscle used as donor nerves instead of the branch to the long head of the triceps [41]. These modifications have been performed through an anterior deltopectoral approach [42] or, alternatively, by selectively reconstructing only the posterior division of the AN [43].

In cases of upper BPI, the thoracodorsal nerve was first proposed in 1990 as a donor nerve to reinnervate chronic traumatic lesions [44]. However, a 2005 analysis demonstrated more favourable outcomes when the thoracodorsal nerve was used to reinnervate the musculocutaneous nerve rather than the AN [45]. More recently, in 2022, the combined use of the thoracodorsal nerve and the branch to the medial head of the triceps was proposed to increase axonal input and improve reinnervation of the AN [46].

Additional donor options include the lower subscapular nerve, as described by Tubbs et al. [47] and subsequently applied clinically in later reports [48,49], as well as the LTN, as reported by Rodríguez-Aceves [50].

Finally, by analogy with the Oberlin technique for restoration of elbow flexion, Haninec and Kaiser proposed transferring fascicles from the ulnar or median nerve to the AN; however, reported outcomes did not exceed M2 on the Medical Research Council (MRC) scale for muscle strength [51].

In 2025, Haninec et al. reported the results of 206 patients undergoing axillary nerve repair, highlighting the versatility of donor nerve selection for AN reconstruction [49].

#### 3.2.2. Extraplexal Nerve Transfer

Several donors outside the injured brachial plexus have been proposed in the literature, and to date no standardized nerve transfer exists. The SAN is one of the commonly proposed donors [34,52,53], followed by the ICNs [34,53,54,55], with at least two ICNs coapted to the AN showing favourable outcomes, and the PH [34]. Nevertheless, intraplexal nerve transfers remain the preferred approach, and when these are not feasible, tendon transfers—such as lower trapezius transfer—are favoured over extraplexal nerve transfer. All data are summarized in Table 2.

## 4. Winging Scapula

### 4.1. Transfer to Long Thoracic Nerve

The LTN, also referred to as the external respiratory nerve of Bell or the posterior thoracic nerve [56], innervates the serratus anterior muscle. Paralysis of this muscle most commonly results in scapular winging. Owing to its superficial course along the lateral chest wall, the LTN is particularly susceptible to injury following falls from height, motor vehicle accidents, and sports-related trauma [57]. Scapular winging may significantly impair shoulder girdle function, subsequently affecting the 3-dimensional use of the affected upper limb. When feasible, surgical reinnervation of the LTN should be considered.

#### 4.1.1. Intraplexal Nerve Transfer

To date, few nerve transfer options have been described for reinnervation of the LTN when direct reconstruction is not feasible. The first reported case of LTN nerve transfer was published by Novak and Mackinnon in 2002, in which the thoracodorsal nerve was used as the donor [58]. Since then, several studies have reported outcomes using the thoracodorsal nerve as a donor, demonstrating satisfactory long-term results with either its posterior or lateral branch [59,60,61,62].

In the same year, Tomaino reported a case demonstrating the feasibility of using the medial pectoral nerve as a donor for the treatment of scapular winging [63]. A combined transfer using both the thoracodorsal and medial pectoral nerves has also been described [64]. However, to date, limited data are available regarding the long-term outcomes of isolated medial pectoral nerve transfer [61].

#### 4.1.2. Extraplexal Nerve Transfer

The available literature on this subject remains limited. The only extraplexal donor described to date is the ICNs, reported in a single patient by Yamada in 2010. In that case, the third and fourth ICNs were used, resulting in complete resolution of scapular winging [65]. A subsequent cadaveric study demonstrated the feasibility of harvesting the fifth and sixth ICNs when the third and fourth had been utilized for musculocutaneous nerve reinnervation [66]. However, no clinical case reports have documented the practical application of this alternative strategy. All data are summarized in Table 3.

## 5. Elbow, Wrist and Finger Extension

### 5.1. Transfer to Radial Nerve

The radial nerve (RN) is a mixed nerve that provides motor innervation to the posterior arm muscles, posterior forearm muscles, and the extrinsic extensors of the wrist and hand, as well as sensory innervation to the skin of the anterolateral arm, distal posterior arm, posterior forearm, and posterolateral aspects of the wrist and hand. It is commonly involved in humeral shaft fractures or may be subject to iatrogenic injury during orthopedic procedures [67]. However, most cases in which nerve transfer is performed to reinnervate the RN involve patients with spinal cord injury (SCI), particularly at the C6 level. Such injuries typically preserve shoulder function and elbow flexion, thereby providing potential donor muscles for reconstructive procedures (Group 2 in the International Classification of Muscle Function in Tetraplegia).

In C6 SCI, function is generally preserved in the brachioradialis, radial wrist extensors, and the supinator muscle, all of which are innervated by motoneurons located rostral to the spinal cord lesion (C5–C6). In contrast, paralysis of the triceps and of the thumb and finger extensor muscles is observed, as their motoneurons are located caudal to the lesion (C7–T1) [68].

#### 5.1.1. Intraplexal Nerve Transfer

##### Elbow Extension

In 2011, Bertelli et al. first proposed the transfer of axillary nerve branches to the teres minor and posterior deltoid to restore elbow extension (triceps brachii function) in patients with SCI [69,70], with an intent opposite to that of Somsak’s procedure. To date, the deltoid-to-triceps nerve transfer is generally preferred over the teres minor–to–triceps transfer, as the posterior deltoid branch can be dissected and directly coapted to a motor branch of the triceps, thereby shortening the reinnervation time [71]. Alternatively, the entire posterior branch of the axillary nerve can be used, while the anterior branch is reserved for cases in which the posterior branch is not intraoperatively stimulable [72]. Regardless of the donor branch selected or the surgical approach (anterior, transaxillary, or posterior), the available evidence indicates that axillary-to-radial nerve transfer represents a reliable option for restoring triceps function, without significant functional deficits at the donor site [73,74].

Another option for triceps reinnervation was proposed in 2015, consisting of the transfer of a terminal motor branch of the ulnar nerve to the flexor carpi ulnaris. This approach has been described as a salvage procedure in cases of extensive BPI with preserved medial cord function [75,76,77,78].

The thoracodorsal nerve was first reported as a donor for triceps reinnervation by Pet et al. in 2011, using an interposed nerve graft [77]. Subsequently, Soldado et al. in 2016 demonstrated the feasibility of direct coaptation in a case series of eight patients, achieving MRC grade M4 strength in all but one patient at a mean follow-up of 21.1 months [79].

Less commonly, the medial pectoral nerve has been used as a donor, as described by Flores in a 2011 case series, both with and without an interposed nerve graft [76], and subsequently proposed again by the same author in 2013 for patients with C5–C7 brachial plexus palsies, with favourable outcomes [80].

Finally, in 2012, Flores proposed a radio-radial nerve transfer for C5–C7 brachial plexus injuries, transferring the fascicle innervating the extensor digitorum communis to a motor branch of the lateral head of the triceps [81]. The safety and efficacy of this technique were later supported by a case series of 13 patients [82].

##### Wrist Extension

Already in 1948, Lurje proposed using a branch of the musculocutaneous nerve to the brachialis muscle to reinnervate the RN in two patients, who recovered some wrist extension [83]. Other nerve transfers reported in the literature include the brachialis-to-ECRL proposed by Fridén [84] and the brachioradialis (RN)-to-ECRL/ECRB proposed by Long Azad [85], both tested only in single case reports. However, the use of a nonsynergistic donor–recipient pair made this approach less favourable and it was eventually abandoned.

Branches of the median nerve for the forearm were subsequently proposed, first by Ustün and colleagues in 2001 [86], and clinically tested by Susan Mackinnon’s group in 2002 [87]. In particular, transferring the branch from the Flexor Digitorum Superficialis (FDS) to the Extensor Carpi Radialis Brevis (ECRB) offers the advantage of delivering regenerating donor axons close to the extensor musculature. In 2012, García-López advocated targeting the Extensor Carpi Radialis Longus (ECRL) instead of the ECRB, citing the greater variability of the ECRB branch and the ease of identifying the ECRL [88]; these results were confirmed in a 2014 case series [89].

To date, most authors have preferred the FDS-to-ECRB transfer [90,91] over the ECRL, as it provides more balanced, centralized wrist extension (inserting into the base of the third metacarpal) and often has a larger, more accessible motor branch [92].

Also in 2012, Bertelli proposed the motor branch of the anterior interosseous nerve (AIN) to the pronator quadratus (PQ) as an alternative for ECRB reinnervation and tested it in 28 patients [93,94] with optimal results later confirmed by subsequent studies [95]. Similarly, García-López proposed the branches supplying the pronator teres (PT) as donor for ECRL in 2014 [89], followed by PT-to-ECRB nerve transfer [96,97].

Finally, to restore wrist extension, the Extensor Carpi Ulnaris (ECU) has also been targeted. Fox et al., in 2015, used the supinator branch of the RN to reanimate this muscle, providing a more functionally useful outcome [98].

##### Finger Extension

Restoration of finger extension, enabling hand opening, was initially attempted through tendon transfers; however, results were often suboptimal [99,100], regardless of the initial level of injury. Active extension of the thumb and fingers is essential for positioning the hand appropriately for grasp and is fundamental for object release. With the advent of nerve transfer techniques, the posterior interosseous nerve (PIN) was identified as the recipient nerve for restoration of this function.

The first donor nerves were selected at the level of the median nerve, including branches to the palmaris longus (PL), flexor carpi radialis (FCR), and FDS [89,101,102] with favourable long-term outcomes reported [103]. Although effective, this strategy has shown limitations in C5–C8 BPI cases [91], and its indication has therefore been largely restricted to injuries selectively involving the radial nerve.

In 2009, Bertelli et al. conducted a cadaveric feasibility study evaluating the transfer of supinator motor branches from the radial nerve to the PIN in cases of C7–T1 lesions [104]. Clinical applicability was subsequently demonstrated in a case series published the following year [99] and the technique was later proposed for use in SCI patients [105]. Since then, several authors have described the supinator-to-PIN transfer, establishing it as a safe and effective procedure for restoring digital extension in both SCI and BPI populations, with comparable success rates [106,107,108,109,110]. Notably, experimental work by Zhang et al. in 2017 demonstrated superior outcomes of distal nerve transfer compared with more proximal neurotization in a rat model [111].

An additional intraplexal donor to consider is the branch to the brachialis muscle from the musculocutaneous nerve, proposed by Palazzi and colleagues in 2006 [112]. However, reported outcomes have been inconsistent, and this technique has not been widely adopted. Most authors have instead preferred to utilize the brachialis branch for alternative reconstructive targets.

##### Extraplexal Nerve Transfer

With regard to extraplexal nerve transfers aimed at restoring radial nerve function, the anatomical location of the available donor nerves generally limits the achievable outcome to elbow extension. The considerable distance between donor and recipient nerves makes restoration of wrist and finger extension unlikely when selective distal branches are targeted. Partial recovery of wrist and finger extension has been reported in cases in which the entire radial nerve trunk was reinnervated rather than specific distal motor branches; however, functional outcomes remain inconsistent and generally inferior compared with those achieved for elbow extension.

Transfer of the SAN has been successfully described for restoration of elbow flexion and shoulder function. Given the critical role of elbow extension in joint stabilization, increasing interest has emerged over the past decades in transferring the SAN to the branch of the radial nerve innervating the long head of the triceps. In 2018, Bulstra et al. described this technique using an interposition autologous nerve graft in a retrospective series of 42 patients [113]. The SAN proved to be an adequate option for restoring elbow extension when other donor nerves were unavailable, despite the relatively prolonged reinnervation time attributable to the length of the required graft [114].

The PH is typically reserved for severe cases in which no other expendable donor nerves are available. Although it is most commonly used to restore elbow flexion, Flores and Socolovsky hypothesized its role in elbow extension reconstruction in 2016 in a series of 10 patients, targeting the radial nerve in seven cases and the branch to the long head of the triceps in three [115]. The modest functional outcomes limited this transfer to highly selected clinical scenarios, preferably targeting only the branch to the long head of the triceps. To further increase the chances of reinnervating the wrist and finger extensors, Lin et al. proposed harvesting the full-length PH via a thoracoscopic approach and subsequently coapting it to the medial portion of the radial nerve at the level of the latissimus dorsi insertion, with favourable results [116].

Another alternative for reinnervation of the long head of the triceps is the use of ICNs, particularly the third, fourth, and fifth ICNs, in cases in which elbow flexion is preserved or has been restored through other nerve transfers [117,118]. The choice of three ICNs rather than two is based on clinical outcomes [54] and on their combined caliber, which more closely matches that of the recipient nerve [119,120]. Several studies have reported favourable results with this strategy, particularly when combined with additional nerve transfers [121,122,123].

Finally, the contralateral C7 (CC7) root has been proposed for restoration of radial nerve function, as first described by Gu et al. in 1992 [124]. Initial approaches targeted the radial nerve trunk, achieving partial recovery of wrist and finger extension [125,126,127,128], whereas subsequent studies focused on the branch to the long head of the triceps [129,130]. However, systematic reviews and meta-analyses have demonstrated that the radial nerve is a less favourable recipient compared with the musculocutaneous nerve [131,132], and that ICNs may provide superior outcomes for elbow extension [133].

In conclusion, a further proposal for radial nerve reconstruction was put forward by Titolo et al. in 2020. In their anatomical study, the peroneal component of the sciatic nerve (common peroneal nerve) was harvested, and the branch to the peroneus brevis muscle was coapted to the branch of the radial nerve innervating the long head of the triceps, whereas the branch to the peroneus longus muscle was connected to the remaining radial nerve trunk [134]. Although technically innovative, this approach has not yet been translated into clinical practice. All data are summarized in Table 4.

## 6. Elbow Flexion

### 6.1. Transfer to Musculocutaneous Nerve

The musculocutaneous nerve (MCN) innervates the three muscles of the arm’s anterior compartment (coracobrachialis, biceps brachii and brachialis) and it is therefore responsible primarily for the elbow flexion and secondary for hand supination. Moreover, it supplies cutaneous innervation through the lateral cutaneous nerve of the forearm in the lateral forearm region. The restoration of elbow flexion is consistently a primary objective in the surgical treatment of traumatic BPI in adults, given its importance for daily activities [135].

#### 6.1.1. Intraplexal Nerve Transfer

The first proposed donors for reinnervation of the MCN were extraplexal, including the SAN and the ICNs. In 1994, however, the group led by Oberlin first proposed harvesting a fascicle from the ulnar nerve as a viable alternative, in what later became known as the “Oberlin procedure” [136]. A key advantage of this technique was sparing the SAN, thereby preserving it for potential transfer to the SSN. Moreover, the close proximity of the ulnar nerve to the motor branch of the MCN innervating the biceps muscle reduces the reinnervation distance and time [137].

Since then, several authors have demonstrated the efficacy and safety of this technique [138,139,140,141,142], particularly when combined with other nerve transfers [143]. It has progressively supplanted nerve graft reconstruction [144,145,146] and more proximal nerve transfers in many settings, although comparative outcomes with extraplexal donors remain controversial in the literature [147,148,149,150,151]. The same procedure was successfully applied to obstetric brachial plexus palsy as early as 2002 by Al-Qattan [152] followed by several other authors, further confirming its versatility [153,154,155,156].

Several modifications of the original technique have been proposed. These include the use of a fascicle from the median nerve instead of the ulnar nerve to minimize potential postoperative functional deficits at the hand level [148,157,158], as well as the combined use of fascicles from both the median and ulnar nerves to enhance elbow flexion strength by reinnervating both the biceps and brachialis muscles—an approach now referred to as the Oberlin II procedure [159,160,161]. In particular, debate persists as to whether the Oberlin II procedure provides superior muscle strength recovery compared with the original Oberlin I technique, with mixed results reported in the available literature [162,163,164,165,166,167].

In 2018, Chepla and Bafus proposed a novel donor, using motor branches to the lower medial head of the triceps and the anconaeus—thus antagonists—to restore elbow flexion in a patient with combined high median, ulnar, and musculocutaneous nerve injury [168]. Although the authors demonstrated technical feasibility, follow-up was limited to less than 3 months, and no data were provided regarding long-term functional outcomes.

Another potential donor for MCN reinnervation is the medial pectoral nerve [169]. Anatomical studies in adults have shown that a nerve graft may be required and that a diameter mismatch may occur between the distal donor and the proximal recipient [170]. Nevertheless, this strategy may represent a valid option in birth-related brachial plexus palsy [171,172].

In 1997, Richardson proposed the use of the thoracodorsal nerve for MCN reanimation [173], offering a novel solution for C5–C6 brachial plexus injuries (BPI), subsequently supported by further studies [45,174,175]. Similarly, in 2007 Haninec described the transfer of the LTN to the MCN, successfully reinnervating three patients [176], thereby suggesting a potential solution for pediatric BPI [177].

More recently, Ferraresi and colleagues described a medial cord-to-MCN transfer using two donor fascicles, primarily those directed to the flexor carpi radialis and flexor carpi ulnaris, and to a lesser extent the flexor digitorum profundus [178]. This technique enables restoration of elbow flexion in multilevel avulsion injuries with at least the T1 root intact, while theoretically preserving the option of a secondary, more distal procedure in the event of initial failure.

Finally, in 2005, an experimental study in cats demonstrated that the LTN and thoracodorsal nerve could be used to neurotize injured contralateral brachial plexus nerves, achieving successful reinnervation [179]. A subsequent cadaveric study in 2009 confirmed the theoretical feasibility of contralateral LTN-to-MCN or SSN transfer [180]; however, to date, no clinical reports of their application have been published.

#### 6.1.2. Extraplexal Nerve Transfer

As early as 1963, Seddon reported the use of ICNs to restore elbow flexion by means of an ulnar nerve graft in a patient with complete BPI [181]. This was followed by several reports over the years [182,183,184,185], addressing optimal timing, prognostic factors [186,187,188], comparisons with other potential donors [149,151,189,190,191,192], the use of ICN transfer in children [193] and long-term outcomes [194]. Overall, ICN-to-MCN transfer has proven effective in restoring elbow flexion with minimal impact on respiratory function. Typically, the third, fourth, and fifth ICNs are harvested, whereas the sixth ICN is used selectively, depending on the surgeon’s preference and intraoperative findings. To date, distal intraplexal neurotizations are preferred over ICN-to-MCN transfer in incomplete BPI [190,195]; however, ICNs remain a valid extraplexal donor option in complete BPI.

The SAN represents the second most frequently used extraplexal donor for elbow flexion reconstruction, owing to its high axonal load and purely motor function. Furthermore, the close functional relationship between the SAN (shoulder elevation) and the MCN (elbow flexion) facilitates postoperative motor re-education, avoiding involuntary muscle activation related to respiratory rhythm, as may occur with ICN or PH transfers [182,196]. In addition, SAN harvesting does not impair diaphragmatic function [197]. Although an interposed nerve graft is generally required [21,198], direct transfer of the SAN to the biceps motor fascicle of the MCN has been described, potentially increasing the likelihood of successful reinnervation [199]. Moreover, the SAN has been reported to be superior to ICNs as a donor for reinnervation in free functional gracilis muscle transfer [200].

In 1996, Gu and Ma first reported the clinical use of the PH for MCN reinnervation in 180 patients, with favourable outcomes [33]. Since then, this technique has become part of clinical practice [201,202,203]. Harvesting can be performed via video-assisted thoracoscopic surgery without the need for a nerve graft [201,204,205] or through a supraclavicular approach combined with an autograft [206]. Nevertheless, its use remains controversial. While some authors report no long-term respiratory complications even after 10 years of follow-up [203,207], others describe a potential reduction in pulmonary vital capacity [208].

The CC7 root may also be employed for MCN reinnervation, particularly in cases requiring simultaneous reconstruction of multiple targets, such as combined CC7-to-MCN and median nerve transfer [151,209], reportedly without negatively affecting median nerve recovery [210]. Despite favourable results, CC7 transfer appears to be inferior to ICN or PH nerve transfer in complete BPI, and to distal neurotization in upper BPI.

Finally, in 1984 Brunelli and Monini proposed the use of cervical plexus branches to restore function in brachial plexus injuries; however, no substantial long-term clinical data supporting this approach are currently available in the literature [211]. All data are summarized in Table 5.

## 7. Hand Pronation, Wrist and Fingers Flexion

### 7.1. Transfer to the Median Nerve

The median nerve (MN) provides motor innervation to the PT, FCR, PL, and FDS. Distal to the medial epicondyle, the AIN arises from the MN and supplies the FDP to the index and middle fingers, the flexor pollicis longus (FPL), and the PQ. More distally, the MN gives rise to the palmar cutaneous branch (PCB) and, within the carpal tunnel, divides into terminal branches including the recurrent motor branch. The latter innervates the thenar musculature—namely the abductor pollicis brevis (APB), opponens pollicis (OP), and the superficial head of the flexor pollicis brevis (FPB)—as well as the first and second lumbricals [212]. Restoration of MN function following direct injury, as well as in cases of lower brachial plexus injury, is therefore critical to reestablish fundamental hand function, particularly finger flexion required for effective grasp.

#### 7.1.1. Intraplexal Nerve Transfer

##### Hand Pronation

Loss of MN function, including the inability to actively pronate the forearm, significantly impairs independence in activities of daily living, such as self-feeding and personal hygiene [213]. Active pronation may be restored by tendon transfer procedures, most commonly described in obstetric BPI, employing the brachioradialis, biceps, supinator, or rerouting of the brachialis muscle. In adult BPI, however, restoration of pronation is often considered a secondary reconstructive goal, as reflected by the limited number of studies addressing its recovery through nerve transfer.

The PT is the primary contributor to forearm pronation [214], and therefore represents the most appropriate target for reinnervation strategies aimed at restoring this function. In 2001, Mackinnon and colleagues reported the first attempt at PT reinnervation using the branch to the FDS, initially in an anatomical study and subsequently in clinical cases [215]. In 2009, the same group described the first ECRB branch–to–PT nerve transfer [216]. Few additional reports evaluating the efficacy of this transfer are available, although overall outcomes appear satisfactory [103].

In their 2025 overview, Maasarani and Wee identified additional potential donor nerves for PT reinnervation, including branches to the brachialis, FCR, and FCU [217]. However, clinical applications remain limited, and the current literature does not allow for a definitive assessment of their effectiveness [218,219,220,221].

##### Wrist Flexion

To the best of our knowledge, no standardized nerve transfer procedure has been established for the restoration of wrist flexion. This function is frequently compensated for by the ulnar nerve–innervated FCU. In cases in which both the median and ulnar nerves are injured, reconstructive priorities are typically directed toward restoration of finger flexion and wrist extension rather than wrist flexion itself.

##### Finger Flexion

Finger flexion is mediated by the FDS and the FDP to the index and middle fingers, together with thumb control provided by the thenar musculature. The principal motor deficits warranting reconstruction involve thumb and index finger flexion, pinch, and grasp. Accordingly, reconstructive strategies primarily target the motor branches to the FDP and FDS, the AIN, and the branches to the thenar muscles.

Thumb opposition, frequently associated with thenar atrophy, may be restored by means of a nerve transfer from the terminal branch of the AIN to the PQ [222,223,224,225]. This approach requires preservation of AIN integrity to be feasible.

An alternative option has been proposed with branches of the RN to restore finger flexion via reinnervation of the FDS, using the distal motor branch of the ECRB, as described by Bertelli et al. in 2012 [226] and reported by Jitpun et al. in 2022 [227], or the supinator branch, as described by Tadisina and Sharma in 2026 [228].

In cases of AIN impairment, the ECRB-to-AIN transfer represents a well-described option [229,230,231,232,233]. Additional reported donor nerves include the brachialis branch to the AIN [71,227,233,234,235,236] and the PT-to-AIN transfer [236,237,238], each associated with favourable outcomes in the literature.

#### 7.1.2. Extraplexal Nerve Transfer

As for other recipients, extraplexal nerve transfers aimed at restoring median nerve function often target the median nerve at its origin or even more proximally at the level of the roots or trunks, as in transfers directed to the lower trunk (C8–T1 roots), resulting in mixed and not always predictable functional outcomes.

One of the most commonly used extraplexal donors is the ICNs, including for reinnervation of the median nerve. Takahashi et al. first proposed this type of transfer in 1991 [239] followed by long-term results reported by Ogino and Naito in 1995 [183], in which two ICNs—usually T5 and T6—were coapted to the proximal arm. Although the transfer was primarily intended for sensory restoration, the authors reported unexpectedly better results in terms of wrist and finger flexion. Similarly, following free muscle transfer for restoration of finger flexion, Doi et al. used the fifth and sixth ICNs for reinnervation, with favourable results [240]. Nevertheless, Jiang et al. compared lower trunk reinnervation in rats using either CC7 or ICNs as donors, demonstrating poorer outcomes with ICNs compared with CC7 [241].

A less frequently used donor is the PH. As in transfers to the radial nerve, its full length is required to obtain a tension-free coaptation without the use of an interposed graft, which can be achieved through a thoracoscopic approach. Moreover, to direct axons preferentially toward the finger flexors, selective neurotization of the posterior fascicular group of the median nerve has been recommended, as demonstrated by Zhao et al. [242,243]. Although only a few reports are available in the literature, overall outcomes appear favourable, with no long-term pulmonary complications reported [244].

First developed by Gu in 1986 [124], the contralateral C7 transfer has been used by several authors over the years for restoration of hand prehension. Results reported in the literature are mixed and difficult to compare because of the high variability in operative approaches [245], including the use of interposition nerve grafts, the choice of recipient nerves, and surgical techniques. Several systematic reviews have attempted to summarize the overall results [132,246], but this transfer remains a valid option when other donors are not available.

Finally, as previously described for the radial nerve, Titolo et al., in their cadaveric study, proposed the ipsilateral peroneal component of the sciatic nerve as a donor. For median nerve reconstruction, they suggested using the branch to the extensor hallucis longus (EHL) and accessory branches to the tibialis anterior for the motor component of the median nerve (medial portion), branches to the extensor digitorum brevis (EDB) and extensor hallucis brevis (EHB) for the anterior interosseous nerve, and terminal sensory branches of the superficial peroneal nerve (SPN) for the distal sensory component of the median nerve [134]. All data are summarized in Table 6.

## 8. Intrinsic Hand Musculature

### 8.1. Transfer to the Ulnar Nerve

The ulnar nerve (UN) provides motor innervation to portions of the forearm and the majority of the intrinsic muscles of the hand. In the forearm, it innervates the flexor carpi ulnaris and the ulnar (medial) portion of the flexor digitorum profundus supplying the fourth and fifth digits. In the hand, it supplies the hypothenar muscles (opponens digiti minimi, abductor digiti minimi, and flexor digiti minimi brevis), the third and fourth lumbricals, the dorsal and palmar interossei, the adductor pollicis, the deep head of the flexor pollicis brevis, and the palmaris brevis.

The critical role of the UN in fine motor control of the hand underscores the importance of timely and effective reinnervation. Accordingly, restoration of ulnar nerve function—similar to that of the MN—represents a primary objective in upper limb reconstruction and functional recovery.

#### 8.1.1. Intraplexal Nerve Transfer

Irreversible degeneration of the distal motor endplates of the intrinsic hand muscles occurs rapidly, often leading to poor functional recovery even after appropriate reconstruction of the UN. This limitation has driven the development of distal intraplexal nerve transfers. The most commonly employed technique is the transfer of the AIN to the UN at the wrist level, which has been shown to be a safe option, requiring sacrifice only of the PQ muscle and effectively mimicking the Martin–Gruber connection while providing a short reinnervation distance. Several authors have reported their long-term outcomes, and although this technique has demonstrated superiority compared with sural nerve grafting [247], some debate persists regarding the optimal transfer configuration.

In particular, some authors have performed a supercharged end-to-side transfer, whereas others have adopted a standard end-to-side approach. The former appears to offer advantages due to the potential for additional reinnervation from the proximal ulnar nerve [248]. Furthermore, Battiston and Lanzetta proposed in 1999 the combination of this transfer with that of the superficial palmar sensory branch of the MN to the motor and sensory components of the UN, with the aim of restoring its sensory function as well [249].

An alternative approach was described by Bertelli et al. in 2019, consisting of an even more distal nerve transfer in which the motor branch to the OP is transferred to the terminal division of the deep branch of the UN [250]. This technique was further evaluated in a case series published in 2024, demonstrating favourable outcomes in terms of reinnervation of the first dorsal interosseous muscle and improvement in pinch strength [251].

Finally, in 2025, Titolo et al. proposed a novel transfer using the MN branch to the PT to restore finger flexion of the fourth and fifth digits in cases of high UN injuries or incomplete brachial plexus injuries, with the aim of improving grip strength [252]. However, this study is currently limited to cadaveric investigation, and no clinical data are available to date.

#### 8.1.2. Extraplexal Nerve Transfer

To the best of our knowledge, with the exception of CC7 nerve transfer—where the ulnar component may be partially involved but is not specifically targeted, as the primary goal is restoration of MN function—the available literature on extraplexal nerve transfers for UN reinnervation remains extremely limited. To date, only an experimental animal study conducted in 1999 has investigated the use of ICNs for UN reinnervation in a feline model [253]. No clinical studies involving human subjects have been reported. All data are summarized in Table 7.

## 9. Nerve Transfer for Extraplexal Nerves

While the primary emphasis is on nerve transfers for brachial plexus reconstruction, it is also important to address transfer strategies targeting the phrenic and spinal accessory nerves, which, although outside the plexus, play a critical functional role.

### 9.1. Transfer to the Phrenic Nerve

The phrenic nerve originates from the anterior rami of the C3, C4, and C5 nerve roots and contains motor, sensory, and sympathetic fibers, providing the sole motor innervation to the diaphragm. In a small proportion of individuals, an accessory PH may be present [254]. Donor PH neurotization was first described in 1948 [255] and has since represented a valid extraplexal donor option for BPI, with several authors reporting no significant long-term impairment of pulmonary function parameters [208,256].

Nevertheless, loss of a single PH results in paralysis of the corresponding hemidiaphragm. Although this condition is often asymptomatic in otherwise healthy individuals, it may represent an additional burden in selected patients, particularly when PH function is lost following traumatic injury, SCI, or in vulnerable populations such as infants, young children, or elderly patients [257].

#### Donors

Although direct repair may be feasible—as demonstrated by Merav et al. in 1983 using a sural nerve graft [258]—Krieger and Krieger were the first to describe nerve transfers to reanimate the diaphragm in patients dependent on long-term positive pressure ventilation due to high cervical SCI [259]. Their technique consisted of an open fourth ICN-to-PH transfer combined with distal PH pacing beyond the site of anastomosis, enabling liberation from prolonged mechanical ventilation. The same procedure may also be performed thoracoscopically in order to reduce surgical morbidity [260].

Jia et al. proposed a nerve transfer using the ninth ICN as a donor to restore PH function after its harvest for BPI reconstruction [261]. However, only a single case was reported, and no objective pulmonary function data were provided, aside from documentation of diaphragmatic movement.

An alternative to ICN transfer was proposed by Yang and colleagues, who employed the SAN in patients with high cervical quadriplegia [262]. This strategy was subsequently adopted by several other authors and has been shown to represent a viable option for ventilator weaning in SCI patients, either in combination with diaphragmatic pacing [263] or without it [264,265].

Finally, PH reinnervation may also be achieved through end-to-side neurorrhaphy between the two phrenic nerves, as demonstrated experimentally by Ding et al. in a rat model [266]. However, to date, no clinical reports have been published. All data are summarized in Table 8.

### 9.2. Transfer to the Spinal Accessory Nerve (XI Cranial Nerve)

Although not a component of the brachial plexus, we elected to include nerve transfers involving this nerve because it is frequently affected by iatrogenic injury during neck surgery, and its dysfunction may result in significant patient morbidity. The SAN innervates the sternocleidomastoid and trapezius muscles [267]. Although the SAN is often considered expendable and is commonly used as a donor nerve in BPI reconstruction, in cases of isolated injury, restoration of SAN function is typically achieved through direct end-to-end coaptation or interposition nerve grafting. However, few reports in the literature describe alternative repair strategies.

#### Donors

The delayed onset of symptoms in isolated SAN palsy may preclude direct coaptation and necessitate the use of long interposition grafts due to stump retraction and diastasis, often resulting in suboptimal functional outcomes. To date, the only reported nerve transfers specifically aimed at restoring SAN function have been described by Goubier and Teboul in 2016 in a single case report, in which the fascicle to the pectoralis major muscle arising from the C7 root was used as the donor [268], and by Mayer et al. in a 2019 case series of five patients [269]. In that study, selective fascicles from the upper trunk directed to the deltoid muscle were used as donor nerves. Outcomes were favourable, with resolution of scapular winging observed in three of five patients. All data are summarized in Table 9.

## 10. Discussion

Over the past decades, nerve transfer surgery has profoundly changed the management of traumatic BPIs, progressively shifting the reconstructive paradigm from proximal nerve grafting toward selective distal neurotization strategies. Improvements in microsurgical techniques, a better understanding of peripheral nerve regeneration, and the increasing ability to identify expendable donor fascicles have considerably expanded the reconstructive options currently available for upper-limb reanimation. As a consequence, modern BPI surgery is no longer based on a limited number of standardized procedures, but rather on a modular and highly individualized reconstructive approach tailored to the pattern of injury, timing of surgery, residual neurological function, and available donor nerves.

Despite this rapid evolution, the literature concerning motor nerve transfers remains highly fragmented. Most reports focus on a single recipient nerve, a specific donor strategy, or a particular clinical scenario, making it difficult to obtain a comprehensive overview of all currently available reconstructive possibilities. Furthermore, emerging techniques are often described only in anatomical studies or isolated clinical reports, and therefore may not yet be incorporated into broader review articles or surgical textbooks. The present review was conceived to address this gap by systematically organizing currently described motor nerve transfers according to the recipient nerve and the functional target to be restored. Rather than comparing the superiority of individual procedures or performing a pooled statistical analysis of outcomes, the aim of this work was to provide a practical and comprehensive map of currently available donor–recipient strategies for upper-limb reinnervation.

One of the most evident trends emerging from the literature is the progressive preference for intraplexal nerve transfers whenever feasible. Intraplexal donors generally provide several advantages, including shorter regeneration distances, more favorable axonal matching, earlier muscle reinnervation, and more physiologic cortical integration. This evolution is particularly evident in the reconstruction of elbow flexion, where distal fascicular transfers such as the Oberlin procedure have largely supplanted more proximal reconstruction techniques and, in many centers, even traditional nerve grafting. Similarly, distal transfers targeting the radial nerve and intrinsic hand musculature have significantly improved the possibility of restoring meaningful distal upper-limb function.

Another major development highlighted by the present review is the increasing distalization and selectivity of nerve transfers. Earlier reconstructive strategies frequently relied on proximal extraplexal donors directed toward major trunks or cords, often requiring long interposition grafts and prolonged regeneration times. In contrast, contemporary techniques increasingly aim to deliver donor axons as close as possible to the target muscle through selective fascicular transfers. Examples include supinator-to-posterior interosseous nerve transfer, anterior interosseous nerve–based procedures for intrinsic hand reinnervation, and selective branch transfers for wrist and finger extension. These approaches reduce the time required for reinnervation and may partially overcome the limited regenerative capacity of distal motor endplates, which historically represented one of the major obstacles in brachial plexus reconstruction.

Nevertheless, extraplexal nerve transfers continue to play a fundamental role, particularly in complete BPIs and root avulsion injuries in which intraplexal donors are unavailable. The spinal accessory, intercostal, phrenic, and contralateral C7 nerves remain essential components of the reconstructive armamentarium. Among these, the SAN continues to represent one of the most reliable donors for restoration of shoulder function, especially through transfer to the suprascapular nerve. ICNs remain valuable donors for restoration of elbow flexion and extension, whereas the PH and contralateral C7 transfer are generally reserved for highly selected cases requiring multiple reconstructive targets. However, compared with distal intraplexal neurotizations, extraplexal transfers are generally associated with longer regeneration distances, greater variability in outcomes, and the potential sacrifice of donor-site function.

The present review also highlights how modern brachial plexus reconstruction has become increasingly “function-oriented.” Rather than reconstructing the plexus anatomically, contemporary strategies prioritize restoration of key functional movements according to their relevance in daily activities. Shoulder stability and abduction, elbow flexion, elbow extension, hand opening, grasp, and intrinsic hand function are approached as separate reconstructive targets that may require distinct donor nerves and different surgical philosophies. Consequently, donor selection has become progressively more strategic. Certain donor nerves are preferentially reserved for specific targets—for example, preservation of the SAN for suprascapular nerve reinnervation or the use of distal fascicular transfers for restoration of elbow flexion and hand opening whenever possible.

Despite the remarkable advances achieved over recent decades, several important limitations remain. Reliable restoration of intrinsic hand musculature continues to represent one of the greatest challenges in peripheral nerve surgery. Even with distal nerve transfers, functional recovery of fine motor control remains inconsistent, likely because of the rapid degeneration of distal motor endplates, the complexity of intrinsic hand muscle coordination, and the limited regenerative capacity of long-distance axonal growth. Similarly, cortical adaptation and motor re-education following nerve transfer remain incompletely understood and may significantly influence functional outcomes independently of technical surgical success.

Another important consideration is the quality and heterogeneity of the currently available evidence. A large proportion of the literature consists of retrospective case series, isolated case reports, or cadaveric feasibility studies. Outcome measures are highly variable, follow-up periods are inconsistent, and direct comparison between studies is often difficult. Moreover, several recently proposed transfers have only been demonstrated anatomically and lack robust clinical validation. Consequently, many of the described procedures should still be considered evolving or experimental techniques rather than established standards of care. For this reason, the purpose of the present review was not to establish superiority among procedures, but rather to summarize and organize the currently available reconstructive possibilities described in the literature.

The present study has several limitations. First, because of the heterogeneity of the included studies, no quantitative analysis or pooled comparison of outcomes was performed. Second, cadaveric studies and preliminary clinical reports were included alongside clinical series, potentially introducing variability in the level of evidence. Third, publication bias likely favored the reporting of technically successful or innovative procedures. Nevertheless, inclusion of both anatomical and clinical studies was considered essential in order to provide a comprehensive overview of the current state of the art and to capture the ongoing evolution of nerve transfer surgery.

Future developments in brachial plexus reconstruction will likely focus on further refinement of selective fascicular surgery, optimization of donor–recipient matching, biologic enhancement of nerve regeneration, and improved postoperative rehabilitation strategies. Advances in neurobiology, intraoperative neurophysiology, and cortical retraining may further improve functional outcomes, particularly for distal targets and intrinsic hand function, which continue to represent the greatest unmet challenge in the field.

## 11. Conclusions

Motor nerve transfers represent an increasingly versatile and evolving group of reconstructive techniques for the management of brachial plexus injuries. The progressive shift toward distal, selective, and function-oriented neurotization has considerably expanded the possibilities for upper-limb reanimation. Intraplexal nerve transfers currently represent the preferred strategy whenever feasible because of their shorter reinnervation distance and more predictable outcomes, whereas extraplexal donors remain indispensable in complete injuries and root avulsions. Rather than relying on a single standardized procedure, modern brachial plexus reconstruction requires a tailored combination of nerve transfers based on the individual injury pattern, available donor nerves, and functional priorities. The present review provides a comprehensive overview of currently described donor–recipient strategies and may serve as a practical reference for surgical planning and future developments in the field.

**Table 10:** Distribution of the included studies among the seven main groups, further subdivided according to the target nerve and donor nerve origin (intraplexal vs. extraplexal). Studies involving the radial and median nerves were additionally categorized based on the functional outcome achieved.

Group	Target Nerve	Type of Transfer	N° Articles	References
Shoulder external rotation and abduction	Suprascapular nerve	Intraplexual transfer	8	[12,13,14,15,16,17,18,19]
		Extraplexal transfer	10	[26,27,28,29,30,31,32,33,34,35]
	Axillary nerve	Intraplexual transfer	14	[38,39,40,41,42,43,44,45,46,47,48,49,50,51]
		Extraplexal transfer	5	[34,52,53,54,55]
Winging scapula	Long thoracic nerve	Intraplexual transfer	7	[58,59,60,61,62,63,64]
		Extraplexal transfer	2	[65,66]
Elbow, wrist and finger extension	Radial nerve	Intraplexual transfer	44	*Elbow extension* [69,70,71,72,73,74,75,76,77,78,79,80,81,82] *Wrist extension* [83,84,85,86,87,88,89,90,91,92,93,94,95,96,97,98] *Finger extension* [99,100,101,102,103,104,105,106,107,108,109,110,111,112]
		Extraplexal transfer	23	[54,113,114,115,116,117,118,119,120,121,122,123,124,125,126,127,128,129,130,131,132,133,134]
Elbow flexion	Musculocutaneous nerve	Intraplexual transfer	46	[45,136,137,138,139,140,141,142,143,144,145,146,147,148,149,150,151,152,153,154,155,156,157,158,159,160,161,162,163,164,165,166,167,168,169,170,171,172,173,174,175,176,177,178,179,180]
		Extraplexal transfer	35	[21,33,149,151,181,182,183,184,185,186,187,188,189,190,191,192,193,194,195,196,197,198,199,200,201,202,203,204,205,206,207,208,209,210,211]
Hand pronation, wrist and fingers flexion	Median nerve	Intraplexual transfer	26	*Hand pronation* [103,215,216,217,218,219,220,221] *Finger flexion* [71,222,223,224,225,226,227,228,229,230,231,232,233,234,235,236,237,238]
		Extraplexal transfer	12	[124,132,134,183,239,240,241,242,243,244,245,246]
Intrinsic hand musculature	Ulnar nerve	Intraplexual transfer	6	[247,248,249,250,251,252]
		Extraplexal transfer	1	[253]
Extraplexal nerves	Phrenic nerve	Not applicable	9	[258,259,260,261,262,263,264,265,266]
	Spinal accessory nerve	Not applicable	2	[268,269]

## Figures and Tables

**Table 1:** All potential donors for suprascapular nerve reinnervation are listed and categorized according to their intraplexal or extraplexal origin. For donors with multiple branches, the specific donor branch(es) are indicated in parentheses.

Potential Donors for Transfer to the Suprascapular Nerve	
Intraplexal	Extraplexal
Dorsal scapular nerve	Ipsilateral spinal accessory nerve
Ipsilateral C7 root (branch for the pectoralis major)	Contralateral spinal accessory nerve
	First intercostal nerve
	Phrenic nerve

**Table 2:** All potential donors for axillary nerve reinnervation are listed and categorized according to their intraplexal or extraplexal origin. For donors with multiple branches, the specific donor branch(es) are indicated in parentheses.

Potential Donors for Transfer to the Axillary Nerve	
Intraplexal	Extraplexal
Radial nerve (branch to long head of the triceps)	Ipsilateral spinal accessory nerve
Radial nerve (branches to medial head of the triceps and to anconeus)	Intercostal nerves
Thoracodorsal nerve	Phrenic nerve
Lower subscapular nerve	
Long thoracic nerve	
Ulnar/median nerve	

**Table 3:** All potential donors for long thoracic nerve reinnervation are listed and categorized according to their intraplexal or extraplexal origin. For donors with multiple branches, the specific donor branch(es) are indicated in parentheses.

Potential Donors for Transfer to the Long Thoracic Nerve	
Intraplexal	Extraplexal
Thoracodorsal nerve	Intercostal nerves (third and fourth)
Medial pectoral nerve	Intercostal nerves (fifth and sixth)

**Table 4:** All potential donors for radial nerve reinnervation are listed and categorized according to their intraplexal or extraplexal origin. Intraplexal donors, given their inherent ability to target specific recipients, are classified according to their primary target. For donors with multiple branches, the specific donor branch(es) are indicated in parentheses.

Potential Donors for Transfer to the Radial Nerve	
Intraplexal	Extraplexal
Elbow extension	Ipsilateral spinal accessory nerve
Axillary nerve	Phrenic nerve
Ulnar nerve (branch to flexor carpi ulnaris)	Intercostal nerves (third, fourth, and fifth)
Thoracodorsal nerve	Contralateral C7
Medial pectoral nerve	Common peroneal nerve (branch to peroneus brevis and peroneus longus)
Radial nerve (branch to extensor digitorum communis)	
Wrist extension	
Musculocutaneous nerve (branch to brachialis)	
Median nerve (branch to flexor digitorum superficialis)	
Median nerve (branch to pronator teres)	
Anterior interosseous nerve (branch to pronator quadratus)	
Radial nerve (branch to supinator)	
Radial nerve (branch to brachioradialis)	
Finger extension	
Median nerve (branch to palmaris longus)	
Median nerve (branch to flexor carpi radialis)	
Median nerve (branch to flexor digitorum superficialis)	
Radial nerve (branch to supinator)	
Musculocutaneous nerve (branch to brachialis)	

**Table 5:** All potential donors for musculocutaneous nerve reinnervation are listed and categorized according to their intraplexal or extraplexal origin. For donors with multiple branches, the specific donor branch(es) are indicated in parentheses.

Potential Donors for Transfer to the Musculocutaneous Nerve	
Intraplexal	Extraplexal
Ulnar nerve (branch to flexor carpi ulnaris)	Intercostal nerves (third, fourth, and fifth)
Median nerve (branch to flexor carpi radialis)	Ipsilateral spinal accessory nerve
Ulnar and median nerve	Phrenic nerve
Radial nerve (branch to medial head of triceps and anconeus)	Contralateral C7
Medial pectoral nerve	Cervical plexus branches
Ipsilateral thoracodorsal nerve	
Ipsilateral long thoracic nerve	
Medial cord	
Contralateral thoracodorsal/long thoracic nerve	

**Table 6:** All potential donors for median nerve reinnervation are listed and categorized according to their intraplexal or extraplexal origin. Intraplexal donors, given their inherent ability to target specific recipients, are classified according to their primary target. For donors with multiple branches, the specific donor branch(es) are indicated in parentheses.

Potential Donors for Transfer to the Median Nerve	
Intraplexal	Extraplexal
Hand pronation	Intercostal nerves (fifth and sixth)
Median nerve (branch to flexor digitorum superficialis)	Phrenic nerve
Median nerve (branch to flexor carpi radialis)	Contralateral C7
Radial nerve (branch to extensor carpi radialis brevis)	Common peroneal nerve (branch to extensor hallucis longus and branches to tibialis anterior)
Musculocutaneous nerve (branch to brachialis)	Common peroneal nerve (branch to extensor digitorum brevis and extensor hallucis brevis)
Ulnar nerve (branch to flexor carpi ulnaris)	
Wrist flexion	
-	
Finger flexion	
Anterior interosseous nerve (branch to pronator quadratus)	
Radial nerve (branch to extensor carpi radialis brevis)	
Radial nerve (branch to supinator)	
Musculocutaneous nerve (branch to brachialis)	
Median nerve (branch to pronator teres)	

**Table 7:** All potential donors for ulnar nerve reinnervation are listed and categorized according to their intraplexal or extraplexal origin. For donors with multiple branches, the specific donor branch(es) are indicated in parentheses.

Potential Donors for Transfer to the Ulnar Nerve	
Intraplexal	Extraplexal
Anterior interosseus nerve (branch to pronator quadratus)	Contralateral C7
Median nerve (branch to opponens pollicis)	
Median nerve (branch to pronator teres)	

**Table 8:** All potential donors for phrenic nerve reinnervation are listed and categorized according to their intraplexal or extraplexal origin. For donors with multiple branches, the specific donor branch(es) are indicated in parentheses.

Potential Donors for Transfer to the Phrenic Nerve
Donors
Intercostal nerve (fourth)
Intercostal nerve (ninth)
Ipsilateral spinal accessory nerve
Contralateral phrenic nerve

**Table 9:** All potential donors for spinal accessory nerve reinnervation are listed and categorized according to their intraplexal or extraplexal origin. For donors with multiple branches, the specific donor branch(es) are indicated in parentheses.

Potential Donors for Transfer to the Spinal Accessory Nerve
Donors
Middle trunk (branch to pectoralis major)
Upper trunk (branch to deltoid)

## Data Availability

No new data were created or analyzed in this study.

## Abbreviations

The following abbreviations are used in this manuscript:

BPIs	Brachial plexus injuries
SAN	Spinal accessory nerve
SSN	Suprascapular nerve
DSN	Dorsal scapular nerve
BPA	Brachial plexus avulsion
AN	Axillary nerve
LTN	Long thoracic nerve
MRC	Medical Research Council scale for muscle strength
ICNs	Intercostal nerves
PH	Phrenic nerve
RN	Radial nerve
SCI	Spinal cord injury
FDS	Flexor digitorum superficialis
ECRB	Extensor carpi radialis brevis
ECRL	Extensor carpi radialis longus
AIN	Anterior interosseous nerve
PQ	Pronator quadratus
PT	Pronator teres
ECU	Extensor carpi ulnaris
PIN	Posterior interosseous nerve
PL	Palmaris longus
FCR	Flexor carpi radialis
CC7	Contralateral C7
MCN	Musculocutaneous nerve
MN	Median nerve
FPL	Flexor pollicis longus
PCB	Palmar cutaneous branch
APB	Abductor pollicis brevis
OP	Opponens pollicis
FPB	Flexor pollicis brevis
EHL	Extensor hallucis longus
EDB	Extensor digitorum brevis
EHB	Extensor hallucis brevis
SPN	Superficial peroneal nerve
UN	Ulnar nerve
